# Intracellular Modulation, Extracellular Disposal and Serum Increase of MiR-150 Mark Lymphocyte Activation

**DOI:** 10.1371/journal.pone.0075348

**Published:** 2013-09-26

**Authors:** Paola de Candia, Anna Torri, Tatiana Gorletta, Maya Fedeli, Elisabetta Bulgheroni, Cristina Cheroni, Francesco Marabita, Mariacristina Crosti, Monica Moro, Elena Pariani, Luisa Romanò, Susanna Esposito, Fabio Mosca, Grazisa Rossetti, Riccardo L. Rossi, Jens Geginat, Giulia Casorati, Paolo Dellabona, Massimiliano Pagani, Sergio Abrignani

**Affiliations:** 1 INGM (Istituto Nazionale Genetica Molecolare), Milan, Italy; 2 Experimental Immunology Unit, Division of Immunology, Transplantation and Infectious Diseases, San Raffaele Scientific Institute, Milan, Italy; 3 Department of Biomedical Sciences for Health, University of Milan, Milan, Italy; 4 Pediatric Clinic 1, Department of Pathophysiology and Transplantation, University of Milan, Fondazione IRCCS Cà Granda Ospedale Maggiore Policlinico, Milan, Italy; 5 NICU and Neonatology, Department of Clinical Sciences and Community Health, University of Milan, Fondazione IRCCS Cà Granda Ospedale Maggiore Policlinico, Milan, Italy; IPMC, CNRS UMR 7275 UNS, France

## Abstract

Activated lymphocytes release nano-sized vesicles (exosomes) containing microRNAs that can be monitored in the bloodstream. We asked whether elicitation of immune responses is followed by release of lymphocyte-specific microRNAs. We found that, upon activation *in vitro*, human and mouse lymphocytes down-modulate intracellular miR-150 and accumulate it in exosomes. *In vivo*, miR-150 levels increased significantly in serum of humans immunized with flu vaccines and in mice immunized with ovalbumin, and this increase correlated with elevation of antibody titers. Immunization of immune-deficient mice, lacking MHCII, resulted neither in antibody production nor in elevation of circulating miR-150. This study provides proof of concept that serum microRNAs can be detected, with minimally invasive procedure, as biomarkers of vaccination and more in general of adaptive immune responses. Furthermore, the prompt reduction of intracellular level of miR-150, a key regulator of mRNAs critical for lymphocyte differentiation and functions, linked to its release in the external milieu suggests that the selective extracellular disposal of microRNAs can be a rapid way to regulate gene expression during lymphocyte activation.

## Introduction

microRNAs (miRNAs) are RNA molecules of 18-25 nucleotides, that regulate gene expression post-transcriptionally via direct interaction with specific sequences present on target mRNAs [1]. miRNAs are present across most species and are highly conserved. More than 2000 miRNAs have been described in the human transcriptome (as for miRBase, Release 19 http://microrna.sanger.ac.uk/) and their control on gene targets has emerged as a critical regulatory principle in mammals, including the immune system [2,3]. It has been demonstrated that miRNAs are present in the extracellular space associated with exosomes [4], which are 20- to 100-nm vesicles (or nanovesicles), formed through the intracellular membrane fusion of multivesicular bodies with the plasma membrane and showing fusogenic activity [5]. The role of exosomes in conveying intercellular communication has been extensively investigated in the immune system [6] as it is the case when dendritic cells internalize exosomes with specific MHC-peptide complexes and in so doing acquire new antigen presenting specificities [7]. It has also been demonstrated that the delivery of exosomes mediates an antigen-driven transfer of miRNAs from the T cell to antigen presenting cells during cognate immune interaction and immune synapse formation [8]. Exosomes are released by most cell types and whence, large amount of miRNAs derived from various tissues/organs are present in human blood and circulate in a cell-free and stable form stabilized by membrane-bound vesicles and protected from endogenous blood RNase activity [9]. Extracellular miRNAs are also carried by membrane-surrounded bodies as large as 1 µm, presumably formed through budding/blebbing of the plasma membrane and generally defined as microvesicles, senescent and apoptotic bodies of similar size. Moreover, it has been observed that circulating miRNAs can travel in blood associated with proteins, and that their preferential association to different biological structures could be dictated by the preferential releasing process of the originating tissue [10].

miRNA expression changes specifically in diseases such as cancer, autoimmunity and viral infections [11-13], making the identification of disease-associated circulating miRNA signatures a possible way of discovering a new class of blood-based non-invasive biomarkers [14]. It has been already shown that circulating miRNA profiles can discriminate healthy subjects from patients affected by cardiovascular diseases, multiple sclerosis, sepsis, liver injury, different tumor types, as well as physiological states, such as pregnancy (reviewed in 15).

The aim of this work has been to investigate the use of circulating miRNAs to monitor the physiological activation of lymphocytes, as the one elicited by vaccination. The work has been developed by first identifying a potential biomarker, miR-150, based on its very high expression in lymphocytes and strong association with vesicles released upon lymphocyte activation *in vitro*. Second, we validated our hypothesis through quantitative assessment of miR-150 in a cohort of serum samples from pandemic flu vaccinated individuals and ovalbumin vaccinated mice, showing an increase of serum miR-150 upon vaccination, which correlates with antibody response.

## Material and Methods

### Vaccination study design and immunogenicity assessment

Vaccinations to adults were administered in the Department of Biomedical Sciences for Health, University of Milan, Italy, during the month of November 2009. Vaccinations to children (aged 6 to 23 months) were administered in the Department of Pathophysiology and Transplantation at Fondazione IRCCS Policlinico Ospedale Maggiore between November 9, 2009, and January 16, 2010 [16]. Among exclusion criteria for children there were any treatment in the previous 4 weeks likely to alter their immune response, previous administration of any influenza vaccine and any acute respiratory tract infection in the 4 weeks before enrolment. Adults received one dose and children two doses (one month apart) of 0.5 ml of MF59-adjuvanted monovalent 2009 pandemic influenza vaccine (Focetria®, Novartis, Siena, Italy), containing 7.5 µg hemagglutinin of A/California/7/2009(H1N1)(X-181). The vaccine was injected into the deltoid muscle (adults) or into the anterolateral part of the left thigh (children). Adult serum was collected at time of enrolment (baseline, T0), and 1 month (25±5 days, T1) after vaccination. Pediatric serum was collected immediately before administration of dose 1 (T0), before administration of dose 2 (28 +/- 2 days after baseline, T1), and 4 weeks later (56 +/- 2 days after baseline, T2). 400 µl of serum from both T0 and T1 of 46 vaccinated adults and 200 µl of serum from T0, T1 and T2 of 50 vaccinated children were used to quantify single miRNAs. In parallel, humoral immune response in both adults and children was assessed by using hemagglutination inhibition test according to standard methods (WHO Manual on Animal Influenza Diagnosis and Surveillance, WHO/CDS/CSR/NCS/2002.5). This assay determined the antibody titers in serum against the hemagglutinin antigens of the 2009 pandemic influenza strain and the antibody titer was expressed as the reciprocal of the highest dilution that inhibited agglutination (European Committee for Proprietary Medicinal Products Note for guidance on harmonization of requirements for Influenza vaccines (CPMP/BWP/214/96). 1997 London: European Agency for the Evaluation of Medicinal Products).

### Purification of Mice and Human Lymphocytes and activation experiments

Untouched CD4^+^ T helper and B lymphocytes were isolated from human peripheral blood mononuclear cells (PBMC), obtained using Ficoll-Paque on buffy coat of healthy donors, using either CD4^+^ T or B lymphocytes isolation kit (Miltenyi Biotec). CD4^+^ T cells were cultured in AIMV medium (devoid of serum, and hence of contaminating miRNAs) and treated with: i) 20 U/ml IL-2 (as un-stimulated control); ii) 100 U/ml IL-2 + 1 µg/ml Phytohemagglutinin (PHA); iii) Phorbol 12-Myristate 13-Acetate (PMA) 25 ng/ml, Ionomycin 1µg/ml iv) 20 U/ml IL-2 + Dynabeads anti-CD3, anti-CD28, 1 bead/cell; v) *Staphylococcus aureus* enterotoxin B (SEB) 2 µg/ml. B lymphocytes were stimulated with 2.5 µg/ml CpG, 5 µg/ml anti-CD40 (gift of Novartis, Siena, Italy) and 10 µg/ml anti-IgM (BD biosciences). At different time points (6, 24, 48, 72 and 96 hours for CD4^+^ and 24 hours for B lymphocytes) cells and conditioned medium were harvested for cell extracts and vesicle isolation (ExoMir kit, Bioo Scientific). Liver and spleen were isolated from 8 weeks old C57BL/6N mice and pressed through a cell strainer to make single-cell suspension. Liver was resuspended in a 40% Percoll solution and mononuclear cells were isolated in the pellet after centrifugation at 1900 rpm. After the lysis of red blood cells, liver mononuclear cells were stained with CD1d tetramer-PE, anti-CD19-FITC and anti-TCRβ-APC antibodies while splenocytes were stained with anti-CD19-FITC, anti-TCRβ-PECy7, anti-CD4-PE and anti-CD8-APC antibodies. A FACS Aria (BD) was used for NKT cell (CD19^-^, CD1d^+^, TCRβ^+^) sorting from liver and for either CD4^+^ (CD19^-^, TCRβ^+^, CD4^+^, CD8^-^) or CD8^+^ (CD19^-^, TCRβ^+^, CD4^-^, CD8^+^) T lymphocyte sorting from spleen. Purified NKT, CD4^+^, CD8^+^ T lymphocytes were cultured separately in AIMV medium and stimulated with PMA 25 ng/ml, Ionomycin 1µg/ml. Cells were collected for RNA extraction before (0 hours) and after (72 hours) activation. Conditioned medium (72 hours) was processed with ExoMir kit for exosome purification.

### Vesicle Preparation

For differential centrifugation, 2 ml of serum diluted to 4 ml in phosphate buffered saline (PBS) were centrifuged to eliminate floating cells (300Xg), dead cells (2,000Xg), cellular debris and apoptotic bodies (serum: 12,000Xg; cell medium: 10,000Xg). The final supernatant was then ultracentrifuged at 110,000Xg (100,000Xg for cell medium) to pellet the nanovesicles. The pellet was then re-suspended in PBS and filtered through a 0.2 micron filter to eliminate residual larger particles, washed in a large volume of PBS, to eliminate contaminating proteins, and centrifuged at the same speed. For microfiltration (ExoMir kit, Bioo Scientific) 0.6-8 ml of cellular medium or 0.4 ml of human serum diluted to 4 ml with PBS were centrifuged at 300Xg and then at 2,000Xg. Supernatants were digested by Proteinase K to eliminate protein complexes and then passed through ExoMir filters. After washing the Top/Bottom filters with 12 ml of PBS (double wash for serum), microvesicles and nanovescicles were separately eluted using 1ml of BiooPure-MP plus ath-mir-159a (final concentration 3 pM).

### miRNA profiling and single miRNA detection by RT-qPCR and Northern Blot

Total RNA from either fresh or frozen human sera and from either cells or centrifuged vesicular pellets was extracted using miRVana miRNA isolation kit (Ambion), as specified in the protocol, with some modifications. Briefly, 400 µl of thawed serum were mixed with 800 µl of lysis solution composed of RNA Lysis Buffer and synthetic ath-miR-159a (final concentration 2.5 pM). This miRNA was used as process control, for technical normalization. RNA extraction from ExoMir Filters was performed as specified in the protocol and RNA was quantified by Ribogreen (Invitrogen), and characterized by Agilent Bioanalyzer. 3 µl of total RNA were processed for Reverse Transcription and Preamplification with Megaplex Primer Pools A v2.1 and B v2.0 (Applied Biosystems), according to manufacturer instruction. TaqMan Low Density Arrays (Applied Biosystems) were run on a 7900HT Fast Real-Time PCR System. A total of 664 human miRNAs, 6 human small RNA and 1 control miRNA from *A. Thaliana* were profiled in parallel. Ct values were extracted using RQ Manager, setting a manual threshold of 0.06. For single miRNA detection, a multiplexed Reverse Transcription reaction (up to 5 miRNAs) was implemented using the TaqMan miRNA Reverse Transcription Kit and miRNA-specific stem-loop primers (Applied Biosystems) according to manufacturer instruction. To profile miRNA expression in human tissues or cultured cells, 10 ng of RNA were processed for RT-qPCR (FirstChoice Human Total RNA Survey Panel, Ambion). DCt values were obtained using the Ct of MammU6 as endogenous control. Serum samples and serum purified nanovesicles were also profiled for 742 miRNAs by using miRNA Ready-to-Use PCR, Human Panel I+II, V2.M RT-qPCR arrays (Exiqon). Normalized values were obtained using a normalization factor resulting from the geometric mean of all expressed miRNAs per sample, i.e. the mean obtained omitting detectors with a Ct >35. For Northern Blotting, we have used 3’ and 5’ Digoxigenin-labeled miRCURY Locked Nucleic Acid (LNA™) microRNA Detection Probes (Exiqon) according to manufacturer instructions.

### Mice studies

MHCII^−/−^ (B6.129-H2Ab1tm1Doi/DoiOrl) and C57BL6/N (Charles River, Italy) were maintained in specific pathogen-free conditions and used at 8 weeks of age. Following collection of pre-immunization sera, four groups of mice were primed at day 0 by subcutaneously (in the left flank) injection of 100 μg/dose of Ovalbumin protein (Sigma) mixed with i) 0.1μg/dose of alpha-galactosylceramide (αGalCer, Alexis), ii) Imject Alum Adjuvant (Pierce, Thermo Scientific), iii) phosphate buffered saline. Blood was then drawn by retro-orbital phlebotomy 7 and 14 days after to determine specific Ig titers of the primary responses on sera. For measurement of antigen-specific antibody titers, individual sera were titrated in parallel at the same time by ELISA and antibody titers are expressed as reciprocal dilutions giving an OD450 > mean blank OD450 + 3 SD. Furthermore, for measurement of circulating miRNAs, 50 µl of sera of pre-vaccinated and 7 and 14 days post-vaccinated mice were processed for total RNA extraction and miR-150 was quantified by single TaqMan assay.

### Ethics Statement

Both the use of biological material (serum and buffy-coat blood) from healthy donors for research purposes and human vaccination studies were approved by the Ethics Committee of IRCCS Ca’ Granda Policlinico Ospedale Maggiore in Milan, Italy. Written informed consent regarding study participation was obtained from all involved adults and the parents or legal guardians of children. All animal procedures were reviewed and approved by the Institutional Animal Care and Use Committee at San Raffaele Scientific Institute.

## Results

### Identification of a miRNA signature associated with nanovesicles in human blood

**Figure 1:**
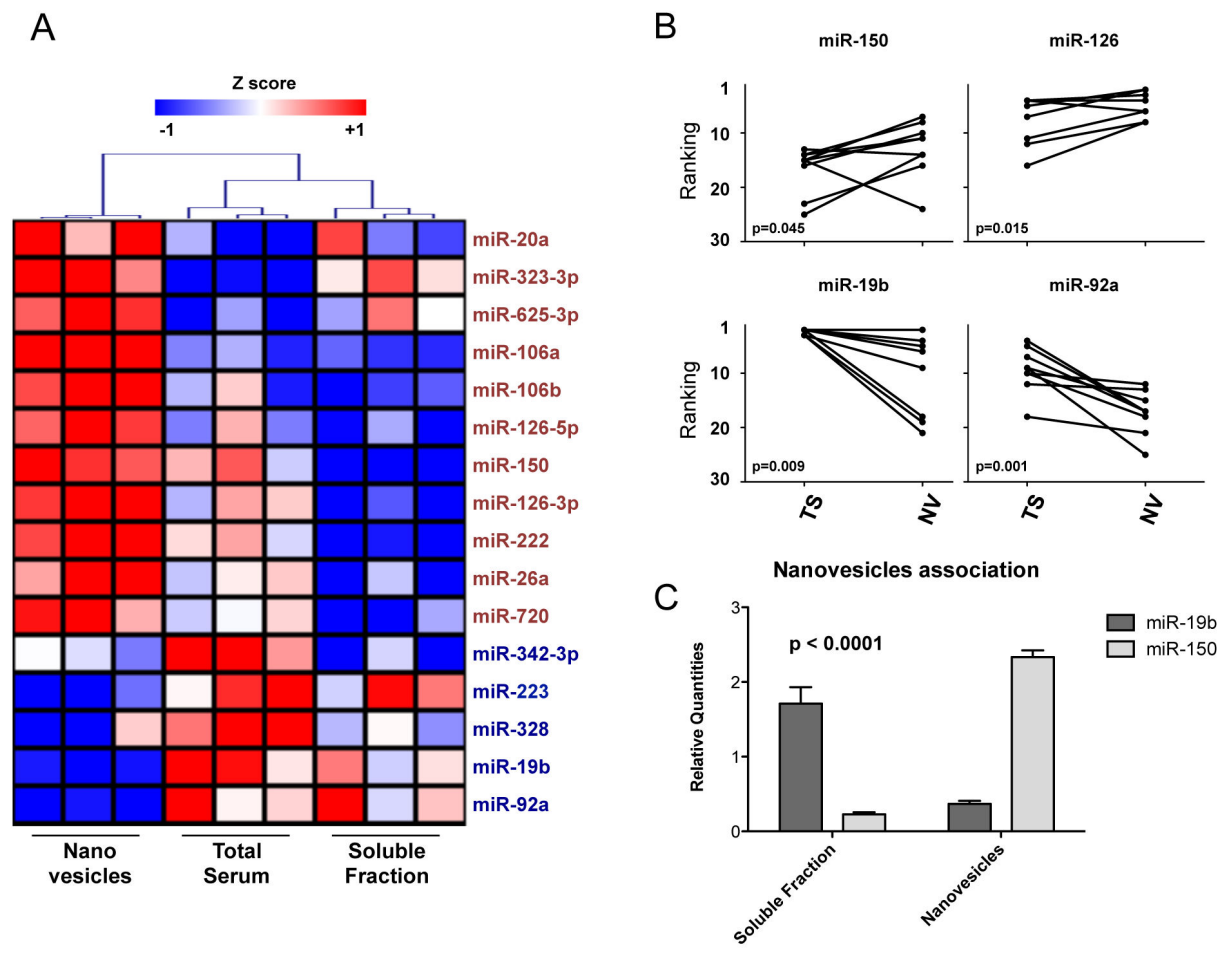
miRNA serum compartmentalization. A. Heatmap for miRNAs significant (p<0.05) upon an ANOVA test (based on F distribution) considering the three reported groups: nanovesicles purified by differential centrifugation (pellet at 110000Xg), total serum and supernatants from the centrifugation at 110000Xg (soluble fraction) from sera of 3 different individuals. Data are representative of two independent experiments. Hierarchical clustering was performed considering Log-transformed normalized relative quantities -Log_10_(NRQs)- of all co-expressed miRNAs with a Ct<35. Distance: Pearson correlation with complete linkage. B. Ranking analysis for miR-150 and miR-126 (upper panels) and for miR-19b and miR-92a (lower panels) in 10 paired samples of total serum (TS) and purified nanovesicles (NV) (7 purified by differential centrifugation and 3 by ExoMiR). Lower ranking position=higher representation. C. miR-19b and miR-150 relatives quantities (2^^-^(^specific compartment Ct – total serum Ct)^) by single RT-qPCR assays in nanovesicles compared to soluble fractions from 3 healthy donors sera (mean of the three samples and SEM are reported) processed by differential centrifugation. p value for a 2-way ANOVA analysis showing an extremely significant effect of serum compartmentalization for different miRNAs is reported.

**Figure 2:**
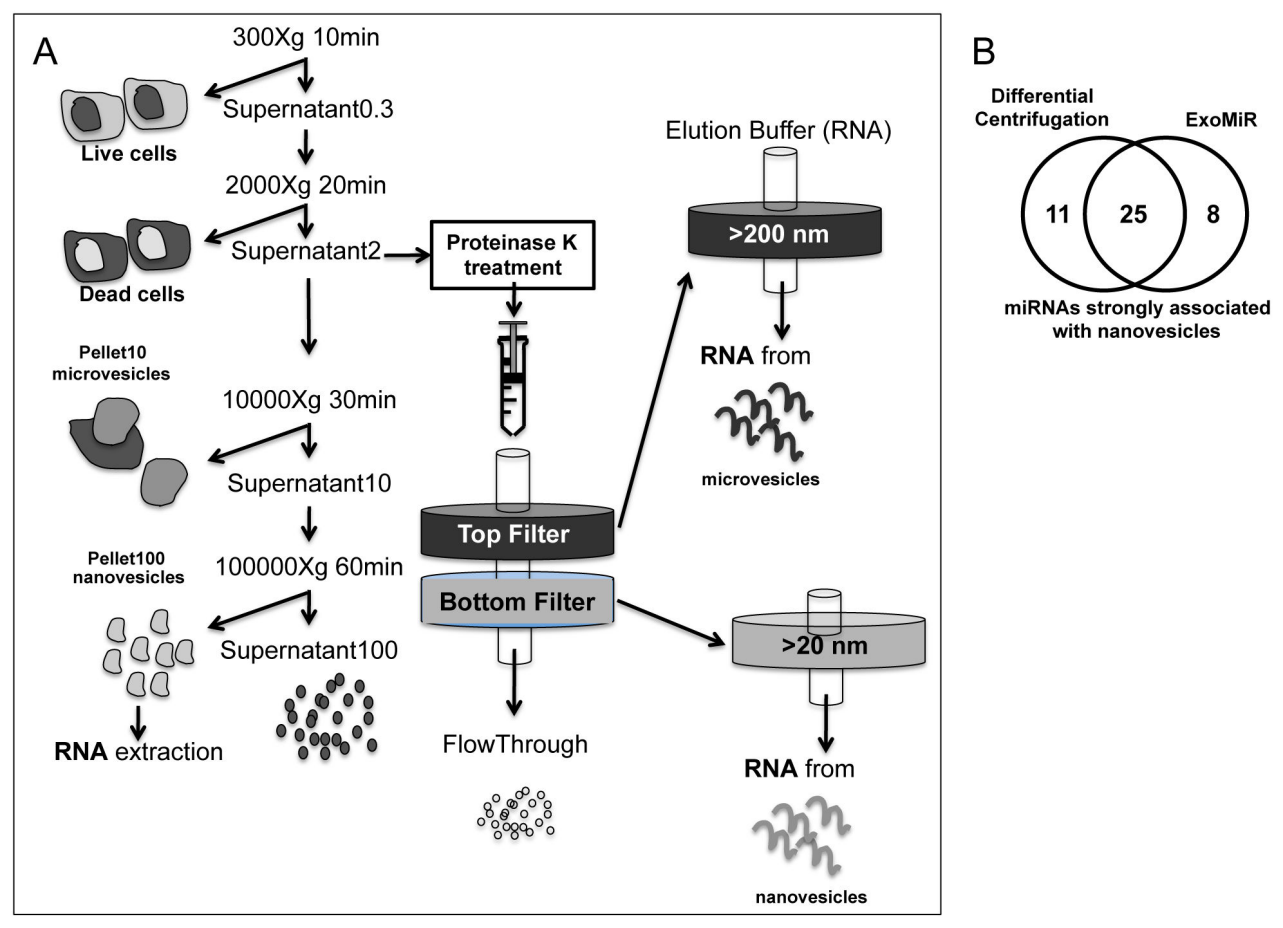
Differential centrifugation and microfiltration for the identification of nanovesicle associated miRNome in human serum. Schematic view of the two nanovesicle purification methods used in this work: differential centrifugation (left) and microfiltration (ExoMir, right). For the latter procedure, serum or cellular medium is passed through 2 filters connected in series. The Top Filter has a larger pore size of approximately 200 nanometers to effectively capture larger particles while the Bottom Filter has a smaller pore size of approximately 20 nanometers for capturing exosomes and other nanovesicles of similar size. The filters are then disconnected and separately flushed by an RNA extraction reagent to lyse the captured particles and release their contents with no preservation of their integrity. B. Venn diagram showing the intersection (25 miRNAs, see list in Table 1) of miRNAs highly expressed (Ct<31 in all samples) in nanovesicles isolated by either differential centrifugation (33 total) or ExoMir (30).

To identify a signature of miRNAs being strongly represented in nanovesicles circulating in human blood, we used TaqMan Low Density Arrays (TLDA) to profile by RT-qPCR the miRNome in serum nanovesicles purified by differential centrifugation, in comparison with the miRNome from serum soluble fraction and total serum. Nanovesicle-associated miRNAs clustered more distantly than miRNAs found in total serum and soluble fraction, suggesting a specific nanovesicular miRNA quantitative profile. Indeed, some miRNAs were found to be enriched in nanovesicles, while others were more represented in total serum and soluble fraction samples (Figure 1A and Table S1 for raw data). The preferential association of specific miRNAs with nanovesicles was also confirmed by ranking analysis and RT-qPCR single assays (Figure 1B,C). We then purified nanovesicles from sera by using ExoMir, an alternative method of purification based on microfiltration. The general workflow for both differential centrifugation [17] and microfiltration [18] is depicted in Figure 2A. By intersecting results of the two purification strategies, we obtained a list of 25 miRNAs that could be regarded as strongly associated with circulating nanovesicles, independently of the purification method. Moreover, 22/25 of these miRNAs were also found consistently expressed by profiling nanovesicular miRNome on a different RT-qPCR platform (Table 1).

**Table 1:** miRNAs strongly associated with nanovesicles circulating in human blood.

**miR-106a**	miR-1274B	**miR-17**	**miR-223**	**miR-320**
miR-106b	**miR-142-3p**	**miR-19a**	**miR-24**	**miR-451**
**miR-126-5p**	**miR-146a**	**miR-19b**	**miR-25**	**miR-720**
**miR-126-3p**	**miR-150**	**miR-20a**	**miR-30b**	**miR-92a**
miR-1274A	**miR-16**	**miR-222**	**miR-30c**	**miR-93**

miRNAs expressed in all samples of nanovesicles (from 6 healthy donors, HD) purified by either differential centrifugation (3 HD) or ExoMir (3 HD) with a Ct<31 in TaqMan Low Density Arrays by Applied Biosystems. Nanovesicles purified by differential centrifugation from 2 independent healthy donors were also profiled by using Exiqon RT-qPCR plates and miRNAs (22/25) that were also detected (Ct<35 in both samples) by this alternative platform are reported in bold.

Remarkably, miR-150, a key regulator of lymphocyte differentiation and functions, was not only part of the signature of miRNAs strongly associated with circulating nanovesicles, but also specifically enriched in this compartment.

### Concomitant intracellular down-modulation and extracellular enrichment of miR-150 in nanovesicles upon lymphocyte activation

**Figure 3:**
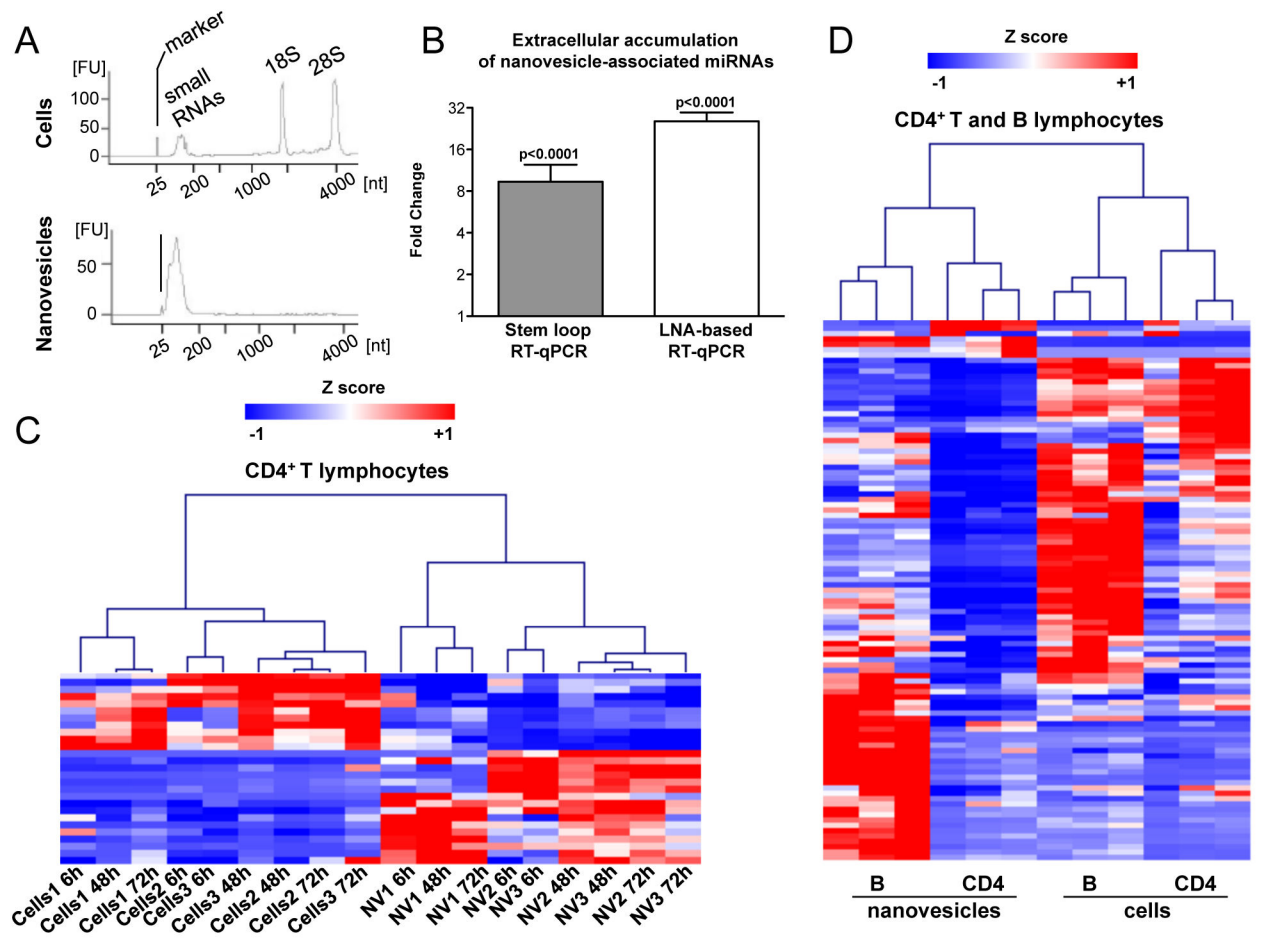
Nanovesicular miRNome of the lymphocyte extracellular milieu is not a mere representation of the intracellular miRNome from parental cells. A. Bio-analyzer qualitative analysis of total RNA extracted 72 hours after activation with Phytohemagglutinin (PHA) from CD4^+^ T lymphocytes (upper panel) and released nanovesicles purified by ExoMir (lower panel). A representative sample is reported. B. Fold change (96 hours compared to 6 hours upon activation with PHA) and SEM of miRNome relative quantities of nanovesicle samples (in biological triplicate) released by CD4^+^ T lymphocyte. Two profiling platforms were used (as indicated, Applied Biosystems Stem-loop RT-qPCR and Exiqon Locked Nucleic Acid (LNA)-based RT-qPCR) and only miRNAs with a Ct<35 were considered. p values of a Wilcoxon matched-pairs signed rank test comparing 6 hours and 96 hours upon activation are reported. C. Hierarchical clustering of Z score values for miRNAs significant (p<0.01) upon a paired t test considering normalized relative quantities of all co-expressed miRNAs with a Ct<35 in CD4^+^ T cellular and nanovesicular compartments (NV) at the indicated time points upon activation with PHA *in* *vitro*. Biological triplicates are reported. Distance for hierarchical clustering on significant miRNA: Pearson correlation with average linkage. D. Hierarchical clustering of Z score values for normalized relative quantities of miRNome from CD4^+^ T and B lymphocytes and their relative released extracellular nanovesicles. Only the 101 co-expressed miRNAs with a Ct<35 were considered. Biological triplicates are reported. Distance: Pearson correlation with average linkage.

**Figure 4:**
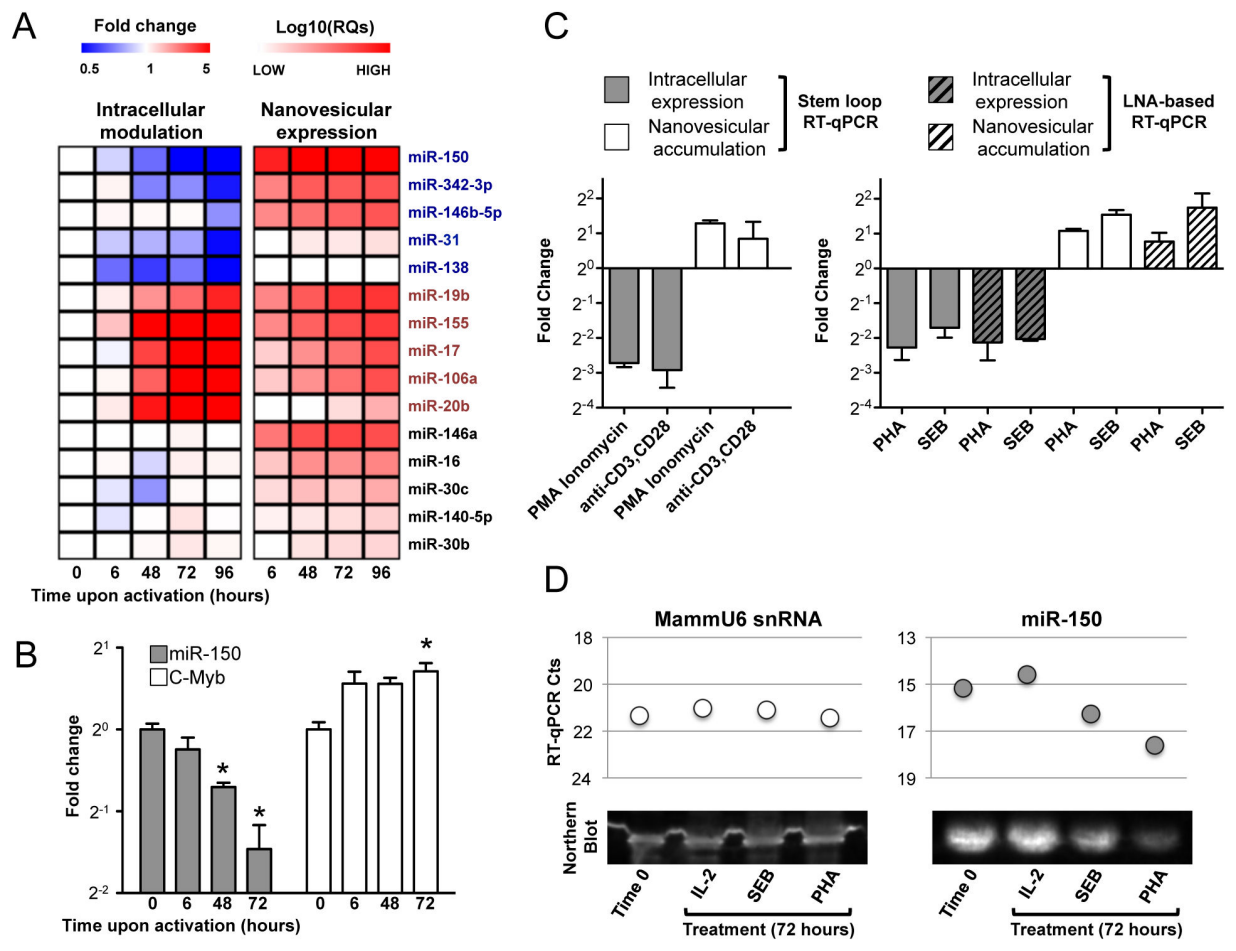
miR-150 intracellular down-modulation and release upon *in vitro* activation of CD4^+^ T lymphocytes. A. Heatmap showing the expression fold change of the indicated miRNAs at the indicated time points upon activation with Phytohemagglutinin (PHA) of CD4^+^ T lymphocytes compared to Time 0 (T0=1) (left panel); and Log_10_ transformed relative expression of the same miRNAs in samples of nanovesicles collected at the indicated time points (right panel). Values are mean of a biological triplicate. The down-regulated (all 5) and the up-regulated (representative 5/56) miRNAs were selected by an ANOVA test (based on F distribution). B. Column chart plotting mean and SEM (of a biological triplicate) of fold change of CD4^+^ T lymphocyte intracellular miR-150 down-regulation and parallel c-Myb up-regulation (normalized by expression of internal control MammU6 and relative to Time 0) at the indicated time points upon activation with PHA. Asterisks indicate a t test resulting in a p value<0.05. C. Column charts plotting mean and SEM (of a biological triplicate) of fold change of CD4^+^ T lymphocyte intracellular miR-150 modulation (normalized by expression of internal control MammU6 and relativized to control, i.e. treatment with IL-2 alone) and nanovesicular accumulation (expressed as relative quantities of extracellular nanovesicular miR-150 upon activation compared to control IL-2 alone treated cells) 72 hours upon starting the indicated treatments (PMA for Phorbol 12-Myristate 13-Acetate; PHA for Phytohemagglutinin; SEB for *Staphylococcus aureus* enterotoxin B). Two profiling platforms were used (as indicated, Applied Biosystems Stem-loop RT-qPCR and Exiqon Locked Nucleic Acid (LNA)-based RT-qPCR) to validate results obtained treating cells with either PHA or SEB (right panel). D. Correspondence between RT-qPCR (upper panel) and Northern Blot (lower panel) for MammU6 snRNA (used as endogenous control, left) and miR-150 (right) expression level at time 0 and 72 hours upon starting the indicated treatments.

In order to identify miRNAs associated with nanovesicles released in the extracellular milieu by human lymphocytes upon activation *in vitro*, primary CD4^+^ T cells were stimulated with Phytohemagglutinin (PHA) and IL-2, while primary B cells were activated with a combination of CpG, anti-CD40 mAb and anti-IgM. Extracellular nanovesicles were then purified from culture media at different time points after activation. Qualitative analysis of total RNA showed a significant enrichment of small RNA molecules in purified nanovesicles compared to cells (Figure 3A). Over time, miRNAs accumulated in the extracellular space, leading to a sharp increase of miRNA relative expression global mean (Figure 3B). Significantly, the extracellular miRNome of primary lymphocytes was not a mere representation of the intracellular one: 27 miRNAs were found to be either up- or down-represented significantly in nanovesicular compared to intracellular compartments of CD4^+^ T lymphocytes (Figure 3C and Table 2). Moreover, the miRNome associated with nanovesicles released by CD4^+^ T and B activated lymphocytes was more similar than the one expressed by the relative parental cells, as shown by clustering analysis (Figure 3D). Similarly to what described in the mouse system [19], in human primary CD4^+^ T lymphocytes miR-150 was significantly down-modulated upon activation. In parallel, we found it to get accumulated in extracellular nanovesicles (Figure 4A and Table S2 for raw data). Ranking analysis also showed miR-150 to be among the most represented miRNAs associated with nanovesicles purified in the extracellular milieu of both CD4^+^ T and B stimulated lymphocytes (Table 3). Intracellular down-modulation of miR-150 upon activation of lymphocytes was paralleled by a slight but significant up-regulation of one of its most relevant targets, c-Myb (Figure 4B). miR-150 intracellular down-modulation and nanovesicular accumulation were dependent on cellular activation, as they were significantly more pronounced in CD4^+^ T lymphocytes activated by different stimuli compared to the same cells growing in IL-2 enriched media as control (fold changes relative to control treatment are shown in Figure 4C). Very similar results were obtained by using three different techniques of miRNA detection (stem-loop RT-qPCR and Locked Nucleic Acid (LNA)-based Universal RT-qPCR, Figure 4C; Northern blot, Figure 4D).

**Table 2:** Differential expression of miRNAs in nanovesicles released by CD4^+^ lymphocytes compared to the intracellular compartment.

**Nanovesicle depleted miRNAs**			
miR-26a	miR-31	miR-142-3p	miR-26b
miR-106a	miR-30c	let-7g	miR-17
miR-30b	miR-146b	miR-20a	
**Nanovesicle enriched miRNAs**			
miR-483-5p	miR-767-3p	miR-151-3p	miR-323-3p
miR-378	miR-618	miR-146a	miR-491
miR-494	miR-138	miR-320	miR-146b-3p
miR-601	miR-181a	miR-186	miR-323-5p

List of miRNAs significantly (p<0.01) either depleted (up) or enriched (down) in nanovesicular compared to intracellular compartment upon a paired t test considering normalized relative quantities of all co-expressed miRNAs with a Ct<35 (Figure 3C).

**Table 3:** miR-150 is among the most represented miRNAs associated with nanovesicles released by human activated lymphocytes.

**T lymph.**	**B lymph.**	**HuH7.5**	**HeLa**
miR-1274B	miR-299-3p	miR-628-5p	miR-1290
miR-1274A	miR-1274B	miR-1274B	miR-1305
**miR-150**	miR-1290	miR-1290	miR-1274B
miR-720	**miR-150**	miR-27a	miR-661
miR-19b	miR-875-5p	miR-518f	miR-1274A
miR-155	miR-661	miR-1274A	miR-720
miR-223	miR-223	miR-520c	miR-483-5p
miR-29a	miR-483-5p	miR-720	miR-601
miR-222	miR-601	miR-299-3p	miR-24
miR-17	miR-422a	miR-661	miR-222

Representation ranking for the ten most represented miRNAs associated with nanovesicles released by the indicated cells after either 72 (CD4^+^ T lymphocytes) or 24 hours of cell culture (B lymphocytes, HuH7.5 and HeLa).

### Serum circulating miR-150 increases after vaccination

**Figure 5:**
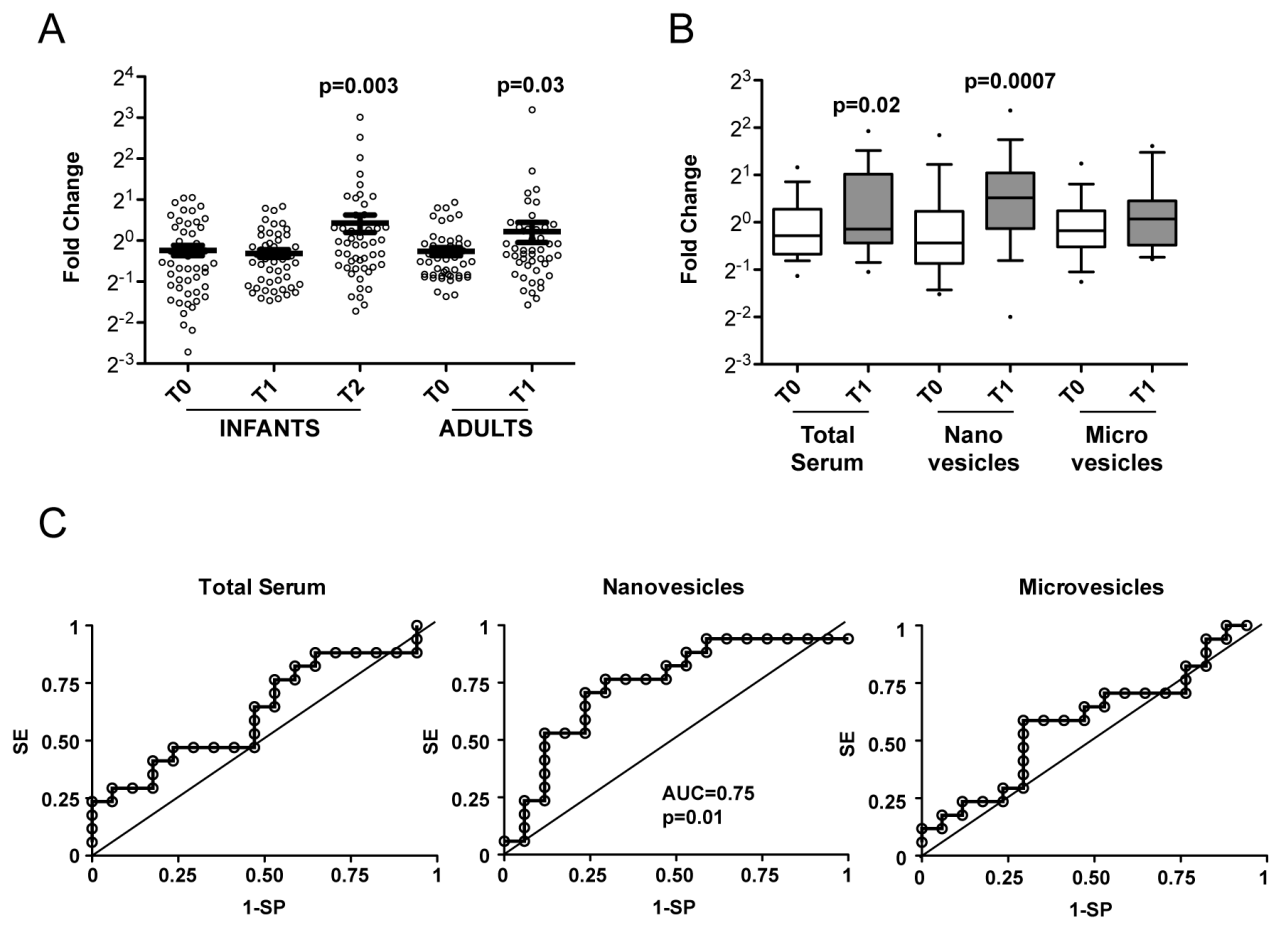
Circulating miR-150 modulation in human serum upon flu vaccination. A. miR-150 quantities relative to exogenous spike-in ath miR-159a in sera of 50 H1N1-MF59 vaccinated children (samples collected at time of first dose, T0, at time of second dose 30 days after, T1 and 30 days after the second dose, T2) (left) and 46 pairs of samples (time of vaccination, T0 and 30 days after, T1) from H1N1-MF59 vaccinated healthy adults (right). Data were centered on the mean at T0 and mean values, SEM and two-tailed paired t test p values are reported. B. Box plot of miR-150 quantities relative to exogenous spike-in ath miR-159a (whiskers: 10-90 percentile) in the indicated serum compartments of 17 pairs of H1N1-MF59 at T0 (white) and T1 (grey). Two-tailed paired t test p values are reported. C. Receiver Operating Characteristic (ROC) curves for total serum, nanovesicular and microvesicular miR-150 increment in H1N1-MF59 vaccinated adults compared to pre-vaccination level (SE=Sensitivity; SP= Specificity). Area under the curve (AUC) and p value (calculated with χ^2^ test) for nanovesicular miR-150 are reported.

Having observed that lymphocytes activated *in vitro* release high quantities of miR-150, and that this miRNA is easily detectable in human serum, we sought to evaluate whether the level of circulating miR-150 would be modulated upon lymphocyte activation *in vivo*, as upon vaccinations. We chose to analyze sera of healthy individuals vaccinated for the influenza virus A/H1N1, the pandemic flu of 2009. The specific vaccine was shown to be immunogenic, safe and well tolerated, being an optimal choice for a starting study. Moreover, the adjuvant MF-59 was already known to evoke greater, long-lasting and broader immune responses [20]. We assessed miR-150 relative quantity by RT-qPCR in serum samples of 50 healthy children and 46 healthy adults before (T0) and 30 days (T1) after immunization with pandemic flu vaccine (for children, all aged <36 months and never vaccinated against influenza, who were administered a second vaccine dose, we had also serum collected 30 days after the second dose, T2). As hypothesized, miR-150 serum level increased after vaccination in adults at T1 and in children after the second dose (T2) (Figure 5A). Differences between the two sample sets are likely due to a partial immunity linked to established memory for seasonal flu that would be present in adults but not in children [21]. miR-1274B, which is strongly associated with vesicles released by lymphocytes as well as by non-lymphoid cells (Table 3), failed to show any modulation in sera of vaccinees (data not shown).

Serum samples from 17 adults vaccinated with H1N1 MF59 (T0 and T1 as above) were also used to purify nanovesicles and vesicles of larger size (between 0.2 and 1 µm, here called microvesicles) using ExoMir. Remarkably, upon vaccination circulating miR-150 increased more significantly in isolated nanovesicles than in total serum, whereas it did not changed in isolated microvesicles, suggesting a specific process of miR-150 release through nanovesicles during immune response (Figure 5B). The Receiver Operating Characteristic (ROC) curves for total serum, nanovesicular and microvesicular miR-150 and the area under the curve (AUC) values confirmed the ability of nanovesicular miR-150 to distinguish serum samples on the basis of vaccination (Figure 5C).

### The increase of serum circulating miR-150 depends on adaptive immune response in humans and mice

**Figure 6:**
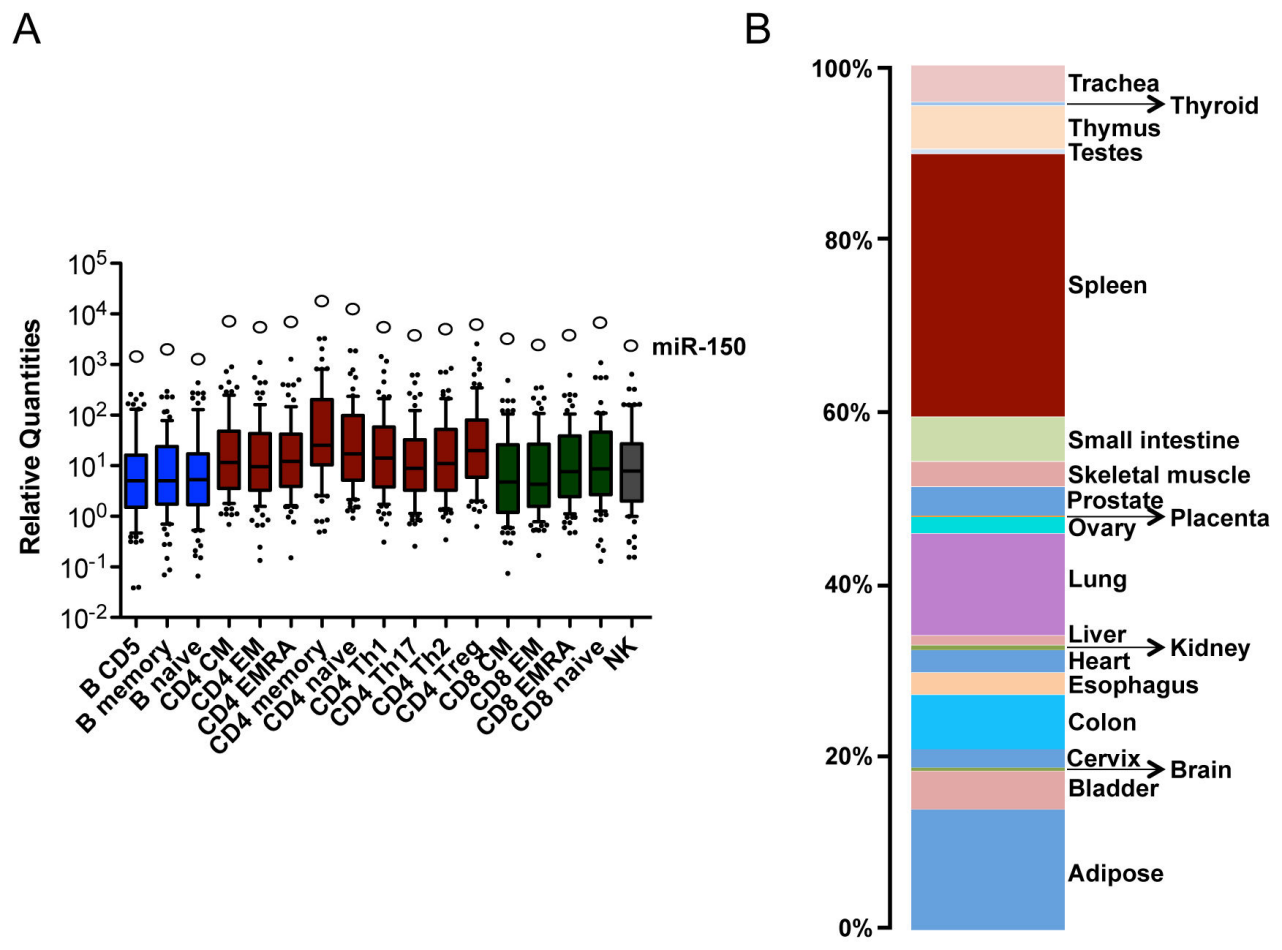
miR-150 expression in human resting lymphocytes and tissues. A. Box plot of miRNome relative quantities in 17 different lymphocyte subsets, as indicated (blue, B; red, CD4^+^ T; green, CD8^+^ T; grey, NK). Only co-expressed miRNAs with a Ct<35 were considered. White circles indicate miR-150 expression level. B. miR-150 level in a panel of 20 different human tissues (as indicated) by RT-qPCR, relative to the internal control MammU6, and reported in percentage relative expression among tissues.

**Figure 7:**
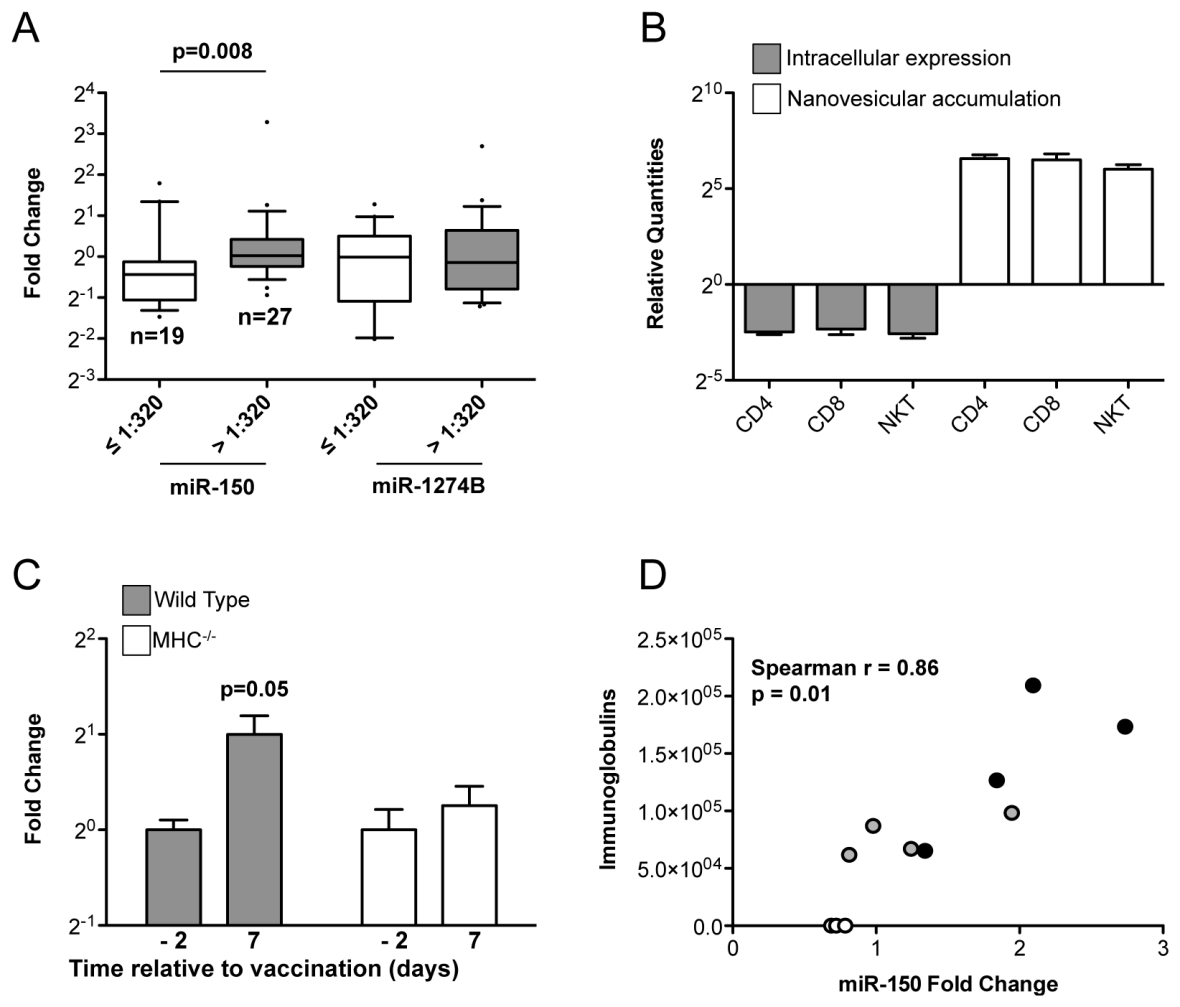
Correlation between circulating miR-150 modulation and immune response. A. Box plot of indicated miRNA quantities at T1 (30 days after vaccination) relative to exogenous spike-in ath miR-159a (whiskers: 10-90 percentile) in 46 flu vaccinated individuals stratified for having developed an antibody response lower (white) or higher (grey) than 1:320, as assessed by hemagglutination inhibition test assay. The p value of a Mann Whitney test is reported. B. Column chart plotting mean and SEM (of a biological triplicate) of mature miR-150 relative quantities in the indicated mouse lymphocytes: intracellular level 72 hours upon activation was normalized first by expression of internal control MammU6 snRNA and then by level at T0. Extracellular accumulation was calculated as 2^-^{Ct(intracellular)-Ct(nanovesicles)}miR-150^ / 2^-^{Ct(intracellular)-Ct(nanovesicles)}MammU6^. Data are representative of two independent experiments. C. miR-150 quantities relative to exogenous spike-in ath miR-159a in wild type and MHCII^-/-^ mice vaccinated with ovalbumin (OVA) adjuvanted with alpha-galactosylceramide (αGalCer) 2 days before vaccination (-2, or T0) and 7 days after vaccination (each treatment normalized to miR mean relative quantity at T0). p value for a paired t test is reported. Four wild type mice and four MHCII^-/-^ mice were used for vaccination experiment. D. Correlation between anti-OVA total Ig concentration (assessed by ELISA) at T=7 days after vaccination in mice vaccinated with αGalCer + OVA (black) or Alum + OVA (grey) and serum circulating miR-150 fold change T1/T0 (T1=7 days after vaccination). Spearman r and p value are reported. miR-150 fold changes values for mice vaccinated with non-adjuvanted OVA are also reported (white).

The observations that miR-150 is both the most highly expressed miRNA in 17 different lymphocyte subsets from peripheral blood mononuclear cells of healthy donors and highly abundant in spleen, lend supports to the evidence that the major source of serum miR-150 are lymphoid cells [(Figure 6 and [22]]. Then, if miR-150 is selectively released by activated lymphocytes, then there could be a correlation between its recordable level and the magnitude of the immune response. Indeed, in flu vaccinated adults, miR-150 serum levels at T1 were significantly higher in individuals mounting higher antibody response (as surveyed by a hemagglutinin inhibition test assay). miR-1274B was added as a control (Figure 7A).

Mouse T lymphocytes showed a pattern of nanovesicular accumulation upon activation *in vitro* similar to the one observed in human lymphocytes, with no major differences between CD4^+^, CD8^+^ or iNKT cells (Figure 7B). Furthermore, consistently with results obtained in vaccinated individuals, mice immunized with ovalbumin (OVA) and alpha-galactosylceramide (αGalCer), an adjuvant leading to a strong activation of lymphocyte response through NKT activation [23], showed a tidy increase of serum miR-150 seven days after vaccination (Figure 7C). When MHCII^-/-^ mice, that are depleted of mature CD4^+^ T lymphocytes [24], were vaccinated in the same way, circulating miR-150 increment was significantly lower in comparison with wild type mice (Figure 7C), confirming the specificity of the observed phenomenon. Moreover, the increment of circulating miR-150 upon vaccination (expressed as fold change T1/T0) was found to significantly correlate with the level of antibodies in wild type mice vaccinated with OVA mixed with either αGalCer or Alum (Figure 7D). In contrast, in mice vaccinated with non-adjuvanted OVA, no antibody production was revealed and circulating miR-150 increase was not detectable after vaccination (Figure 7D). These results together strongly suggest that the increment in the level of circulating miR-150 upon immunization is lymphocyte-derived and dependent on the magnitude of immune response.

## Discussion

Expression of intracellular miRNAs and their target genes have been extensively studied in activated CD4^+^ T cells [3,25]. For instance, down-regulation of mouse miR-150 upon activation [26] associates with up-regulation of its target c-Myb, a transcriptional factor that promotes lymphocyte survival by inducing the pro-survival protein Bcl2 [27]. We report here that upon activation of both human and mouse primary lymphocytes, there is a reduction of miR-150 intracellular expression concomitantly with an extracellular accumulation of exosome-associated mir-150. We speculate that the release of miR-150 rich vesicles may represent a new and additional layer of regulation of miR-150 expression level and, in turn, of the mRNAs that are targeted by this miRNA and critically control lymphocyte responses.

It has been recently shown that reduced miR-150 serum concentrations are associated with an unfavorable outcome in critically ill patients with sepsis and it has been hypothesized that lower level of circulating miR-150 might lead to a de-repression of genes such as CXCR4 and c-Myb, that are linked to immune response activation and poor prognosis [28]. In line with this hypothesis, it is tempting to speculate that the increase of circulating miR-150 level that we observe upon vaccination may be part of a negative regulatory loop aimed at down-modulating adaptive immune responses through the transmission of extracellular messages to other immune cells and the consequent regulation of miR-150 target genes. This possibility is not unprecedented as the delivery of miRNAs by transfer through nanovesicles is a recently described mechanism of cell-cell communication, potentially occurring at a distance [29]. In particular, it has been demonstrated that vesicle-packaged miR-150 specifically regulates target gene expression and function in recipient cells [30]. Furthermore, an RNA-based paracrine mode of signal transmission may be also well suited in controlling space-confined processes, such as initiation and development of adaptive immune responses in secondary lymphoid organs. The transfer of proteins and nucleic acids such as small RNA between T cells and antigen-presenting cells has indeed been described, involving exchange of extracellular vesicles during the formation of the immunological synapse [8]. As nanovesicles can deliver multiple miRNAs, functionally related genes could be suppressed simultaneously, leading to a very effective control over neighboring cells [31]. Hence, a characterization of cellular targets of the above miRNAs may provide new insight into functionally relevant biological patterns of immune cells, which could be targeted by new immune-modulatory pharmacological approaches.

During immune response, it is likely that miRNAs are released into the surrounding environment via active release of nanovesicles by activated lymphocytes [6]. Here we describe a specific modulation of circulating miR-150 upon immunization, more evident in purified nanovesicles compared to total serum, that is suggestive of a phenomenon of massive release of nanovesicle associated miRNAs occurring *in vivo* during the physiological activation of the immune system. Hence our study supports the hypothesis that analyzing miRNA levels in serum is a promising area for identifying new biomarkers of immune response and that purification of nanovesicles may be a useful step to enrich for miRNAs segregating to specific structures, and increase sensitivity for the immune response.

miR-150 is highly expressed in all lymphocyte populations, hence it is a very general sensor of adaptive immune response but our study also suggests that different lymphocyte populations may display significantly different selective enrichment of specific extracellular miRNAs. Thus, upon their identification, it would be virtually possible to mark the elicitation of different lymphocyte populations through the assessment of their specific serum signatures [32]. The identification of these signatures may then become an innovative tool to provide pivotal information about the nature of the immune response in diseases involving the immune system or during clinical trials with new vaccines, adjuvants and immune-modulatory drugs.

## Supporting Information

Table S1 **miRNA serum compartmentalization.** Raw data (Ct values) for miRNAs profiled by Applied Biosystems Stem-loop RT-qPCR from the three reported groups: nanovesicles purified by differential centrifugation (pellet at 110000Xg), total serum and supernatants from the centrifugation at 110000Xg (soluble fraction) from sera of 3 different individuals. Data are from one of two independent experiments that gave comparable results.
(TXT)

Table S2 **Nanovesicular miRNome of the lymphocyte extracellular milieu is not a mere representation of the intracellular miRNome from parental cells.** Raw data (Ct values) for miRNAs profiled by Applied Biosystems Stem-loop RT-qPCR from CD4^+^ T cellular and nanovesicular compartments at the indicated time points upon activation with PHA *in* *vitro*. Biological triplicates are reported. Following, miRNome from B lymphocytes and their relative released extracellular nanovesicles at the indicated time point of activation.
(TXT)
